# Anxiety and depression prevalence and their risk factors in lupus nephritis patients: A case–control study

**DOI:** 10.1002/iid3.689

**Published:** 2022-08-22

**Authors:** Ying Hu, Ge Zhan

**Affiliations:** ^1^ Department of Nephrology, the First Affiliated Hospital of Harbin Medical University Harbin China; ^2^ Department of General Therapy The First Specialized Hospital of Harbin Harbin China

**Keywords:** anxiety, depression, lupus nephritis, prevalence, risk factors

## Abstract

**Introduction:**

Anxiety and depression exhibit a high prevalence in systemic lupus erythematosus (SLE) patients, while this issue is seldom explored in lupus nephritis (LN). Hence, the current study aimed to investigate the prevalence of anxiety and depression, and the risk factors for these mental disorders in LN patients.

**Methods:**

Fifty LN patients, 50 non‐LN SLE patients, and 50 health control (HCs) were enrolled. The Hospital Anxiety and Depression Scale (HADS) for anxiety (HADS‐A) score and HADS for depression (HADS‐D) score were evaluated.

**Results:**

HADS‐A score was highest in LN patients (median 7.0, interquartile range [IQR]: 6.0–10.0), followed by non‐LN SLE patients (median 6.0, IQR: 5.0–8.0), and lowest in HCs (median 5.0, IQR: 3.0–7.0) (*p* < .001). Besides, the anxiety rate was most frequent in LN patients (38.0%), followed by non‐LN SLE patients (28.0%), least common in HCs (12.0%) (*p* = .011). HADS‐D score was highest in LN patients (median 7.5, IQR: 6.0–11.0), followed by non‐LN SLE patients (median 6.0, IQR: 5.0–8.3), and lowest in HCs (median 4.0, IQR: 2.0–6.3) (*p* < .001). Similarly, the depression rate was most prevalent in LN patients (50.0%), subsequently the non‐LN SLE patients (30.0%), and rarest in HCs (10.0%) (*p* < .001). Furthermore, in LN patients, age (*p* = .009), LN activity index (*p* = .020), alopecia (*p* = .023), 24 h proteinuria (*p* = .044), and C‐reactive protein (*p* = .049) were independently correlated with higher anxiety risk; meanwhile, age (*p* = .001) and LN activity index (*p* = .009) were independently correlated with higher depression risk.

**Conclusion:**

Anxiety and depression are highly prevalent, which link to aging, alopecia, inflammation, and severe renal involvement in LN patients.

## Graphical abstract

1. Anxiety and depression are highly prevalent in lupus nephritis (LN) patients.
2. Anxiety links with aging, LN activity index, alopecia, 24 h proteinuria, and C‐reactive protein in LN patients.
3. Depression links with aging and LN activity index in LN patients.

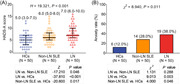

## 1 INTRODUCTION

Lupus nephritis (LN), a severe complication of systemic lupus erythematosus (SLE), could result in renal dysfunction and increased mortality risk in SLE patients.^1^, ^2^ Currently, the treatment of LN mainly includes induction therapy of cyclophosphamide, mycophenolate mofetil/mycophenolate acid (MMF/MPA), and calcineurin inhibitors (CNI), as well as subsequent maintenance therapy by MMF/MPA, azathioprine (AZA), and so forth.^3^, ^4^, ^5^, ^6^ However, despite the advance in treatment approaches in decades, the kidney failure risk still remains unacceptably high; notably, approximately 30% of LN patients would finally develop end‐stage renal disease (ESRD).^7^, ^8^ Apart from that, the LN patients might also suffer from mental disorders (such as anxiety, depression, suicidality, etc.), which further prolong the LN treatment duration and affect patients' quality of life.^9^

Several clinical studies have investigated the prevalence of anxiety and depression in SLE patients. One study discloses that the prevalence of anxiety and depression is 44.0% and 36.0%, respectively in SLE patients.^10^ Besides, another study reveals that the frequency of anxiety and depression is 34.0% and 51.0%, respectively in SLE patients.^11^ What's more, it is illustrated that the anxiety and depression rate of SLE patients is much higher compared to health controls (HCs).^12^, ^13^ However, the research about the clinical relevance of anxiety and depression with LN is limited. Herein, the current study enrolled 50 LN patients, 50 non‐LN SLE patients, and 50 HCs, then assessed the hospital anxiety and depression scale (HADS) score among them, which aimed to investigate the prevalence of anxiety and depression, and their risk factors in LN patients.

## 2 MATERIALS AND METHODS

### 2.1 Patients

The case‐control study included 50 LN patients who were admitted to hospital between May 2018 and March 2021. The screening criteria were: (a) diagnosed as LN according to the American College of Rheumatology criteria^14^; (b) aged over 18 years; (c) volunteered to participate in the study. Patients with one of the following conditions were ineligible: (a) had a history of hematologic malignant disease or cancer; (b) ongoing pregnancy or lactating female patient. The study also enrolled 50 SLE patients without LN (non‐LN SLE patients) who were admitted to the hospital from May 2018 to March 2021 as disease controls, as well as 50 health subjects who came to hospital for medical examination during the same period as HCs. The non‐LN SLE patients, as well as HCs who had ongoing pregnancy, hematologic malignant disease, or cancer, were excluded. The study protocol was approved by Ethics Committee. All subjects signed the informed consents.

### 2.2 Data collection

Clinical characteristics were collected from LN patients after enrollment, which included age, gender, disease duration, systemic lupus erythematosus disease activity index (SLEDAI) score, clinical manifestations, LN classification, LN activity index, LN chronicity index, and biochemical indexes. SLEDAI was scored for the measurement of disease activity in LN patients.^15^ LN classification was categorized into six classes according to International Society of Nephrology/Renal Pathology Society (ISN/RPS) 2003 classification criteria, and supported by renal biopsy.^16^ Besides, the LN activity index (0–24 points) as well as LN chronicity index (0–12 points) were scored based on the method proposed by Austin et al.^17^

### 2.3 Evaluation

All eligible subjects received Hospital Anxiety and Depression Scale (HADS) assessment after enrollment. The HADS for anxiety (HADS‐A) score was used for evaluation of anxiety status, and HADS‐A score >7 was considered as anxiety. The HADS for depression (HADS‐D) score was applied for assessment of depression status, and HADS‐D score >7 was considered as depression.^18^

### 2.4 Statistics

Statistical analysis was fulfilled by SPSS (version 22.0, IBM Corp.), and graphs were generated by GraphPad Prism (version 7.02, GraphPad Software Inc.). Kolmogorov–Smirnov test was carried out for determination of the data distribution. The comparison of HADS‐A and HADS‐D scores among groups were determined by the Kruskal–Wallis H rank sum test; the comparison of anxiety and depression rates among groups was determined by the Chi‐square test. The factors related to anxiety and depression were assessed using logistic regression analysis, and all factors were included in multivariate models with step‐forward methods. *p* < .05 was considered statistically significant.

## 3 RESULTS

### 3.1 Clinical characteristics of LN patients

The mean age was 46.3 ± 16.7 years in LN patients, among which there were 7 (14.0%) males and 43 (86.0%) females (Table 1). In terms of disease features, the median disease duration was 55.5 (interquartile range [IQR]: 2.0–122.0) months; meanwhile, the median SLEDAI score was 9.0 (IQR: 6.0–15.8). Regarding LN classification, 5 (10.0%), 6 (12.0%), 22 (44.0%), 5 (10.0%), 4 (8.0%), and 8 (16.0%) patients were diagnosed with Class II, Class III, Class IV, Class V, Class V + III, and Class V + IV, separately. More detailed information was listed in Table 1.

**Table 1:** Clinical characteristics of LN patients.

Items	LN patients (*N* = 50)
Demographics	
Age (years), mean ± SD	46.3 ± 16.7
Gender, *n* (%)	
Male	7 (14.0)
Female	43 (86.0)
Disease features	
Disease duration (months), median (IQR)	55.5 (2.0–122.0)
SLEDAI score, median (IQR)	9.0 (6.0–15.8)
Histological examinations	
LN classification, *n* (%)	
Class II	5 (10.0)
Class III	6 (12.0)
Class IV	22 (44.0)
Class V	5 (10.0)
Class V + III	4 (8.0)
Class V + IV	8 (16.0)
LN activity index, median (IQR)	8.0 (5.0–10.0)
LN chronicity index, median (IQR)	3.0 (2.0–4.0)
Biochemical indexes	
WBC (×10^9^/L), median (IQR)	5.54 (4.16–8.06)
ALB (g/L), mean ± SD	27.8 ± 6.8
ALT (U/L), median (IQR)	17.5 (11.0–33.8)
24 h proteinuria (g), median (IQR)	1.77 (0.32–3.54)
Scr (µmol/L), median (IQR)	82.5 (66.2–108.0)
C3 (g/L), median (IQR)	0.54 (0.38–0.77)
C4 (g/L), median (IQR)	0.14 (0.08–0.17)
IgA (g/L), mean ± SD	2.16 ± 0.85
IgG (g/L), median (IQR)	13.77 (9.20–19.75)
IgM (g/L), median (IQR)	0.78 (0.60–1.39)
ANA positive, *n* (%)	48 (96.0)
Anti‐dsDNA positive, *n* (%)	27 (54.0)
Anti‐Sm positive, *n* (%)	11 (22.0)
Anti‐SSA positive, *n* (%)	35 (70.0)
Anti‐SSB positive, *n* (%)	6 (12.0)
Anti‐nRNP positive, *n* (%)	23 (46.0)

Abbreviations: ALB, albumin; ALT, alanine aminotransferase; ANA, Anti‐nuclear antibody; Anti‐dsDNA, anti‐double‐stranded DNA antibody; anti‐Sm, anti‐Smith antibody; anti‐SSA, anti‐Sjögren's‐syndrome‐related antigen A antibody; anti‐SSB, anti‐Sjögren's‐syndrome‐related antigen B antibody; anti‐nRNP, anti‐nuclear ribonucleoprotein antibody; BUN, blood urea nitrogen; C3, complement component 3; C4, complement component 4; CRP, C‐reactive protein; ESR, erythrocyte sedimentation rate; Hb, hemoglobin; IgA, immunoglobulin A; IgG, immunoglobulin G; IgM, immunoglobulin M; IQR, interquartile range; LN, lupus nephritis; Scr, serum creatinine; SD, standard deviation; SLEDAI, systemic lupus erythematosus disease activity index; WBC, white blood cell.

### 3.2 HADS score, anxiety, and depression rate among HCs, non‐LN SLE, and LN patients

**Figure 1:**
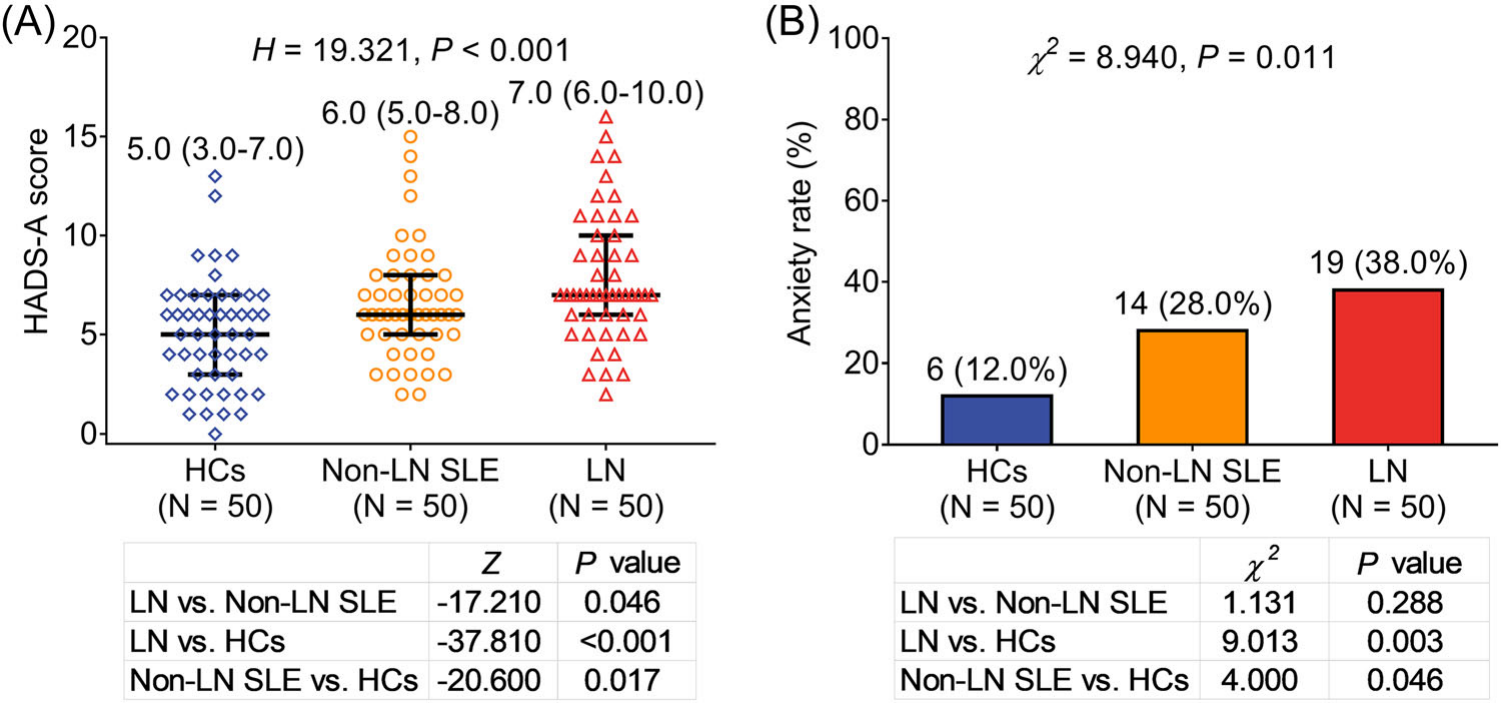
Comparison of anxiety status among HCs, non‐LN SLE, and LN patients. Comparison of HADS‐A score (A) and anxiety rate (B) among HCs, non‐LN SLE, and LN patients. HADS, Hospital Anxiety and Depression Scale; HCs, health controls; LN, lupus nephritis; SLE, systemic lupus erythematosus disease

HADS‐A score was highest in LN patients (median 7.0 [IQR: 6.0–10.0]), followed by non‐LN SLE patients (median 6.0 [IQR: 5.0–8.0]), and lowest in HCs (median 5.0 [IQR: 3.0–7.0]) (*H* = 19.321, *p* < .001, Figure 1A). Besides, anxiety rate was also highest in LN patients (38.0%), followed by non‐LN SLE patients (28.0%), and lowest in HCs (12.0%) (*χ* ^*2*^ = 8.940, *p* = .011, Figure 1B).

**Figure 2:**
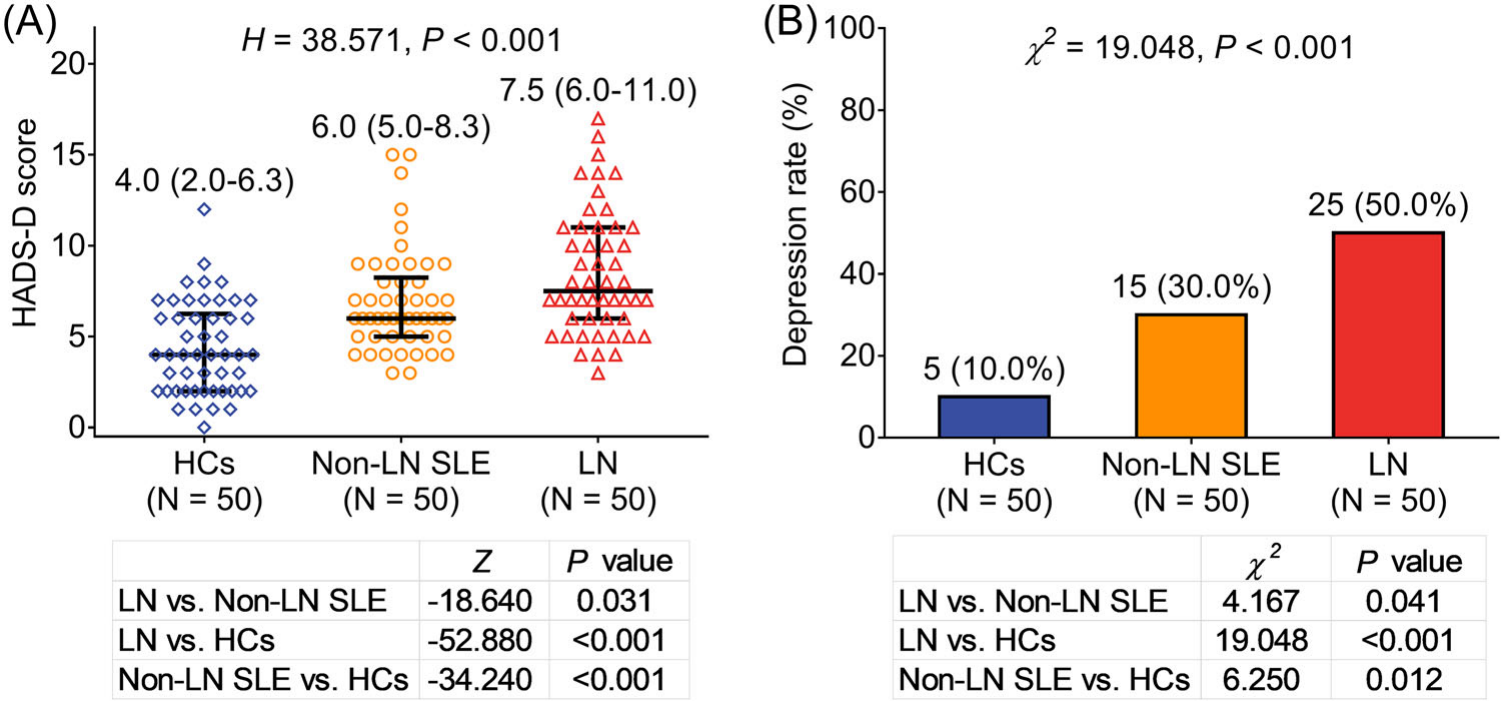
Comparison of depression status among HCs, non‐LN SLE, and LN patients. Comparison of HADS‐D score (A) and depression rate (B) among HCs, non‐LN SLE, and LN patients. HADS, Hospital Anxiety and Depression Scale; HCs, health controls; LN, lupus nephritis; SLE, systemic lupus erythematosus disease

HADS‐D score was highest in LN patients (median 7.5 [IQR: 6.0–11.0]), followed by non‐LN SLE patients (median 6.0 [IQR: 5.0–8.3]), and lowest in HCs (median 4.0 [IQR: 2.0–6.3]) (*H* = 38.571, *p* < .001, Figure 2A). Similarly, depression rate was also highest in LN patients (50.0%), followed by non‐LN SLE patients (30.0%), and lowest in HCs (10.0%) (*χ* ^*2*^ = 19.048, *p* < .001, Figure 2B).

### 3.3 Factors relating to anxiety risk in LN patients

Univariate logistic regression disclosed that age (odds ratio [OR] = 1.051, 95% confidence index [CI]: 1.011–1.093, *p* = .012), SLEDAI score (OR = 1.127, 95%CI: 1.027–1.237, *p* = .012), affected nervous system (Yes vs. No) (OR = 6.788, 95%CI: 1.516–30.392, *p* = .012), alopecia (Yes vs. No) (OR = 3.361, 95%CI: 1.016–11.117, *p* = .047), LN classification (Class IV [with or without V] vs. Class III [with or without V], II, or V) (OR = 6.476, 95%CI: 1.563–26.836, *p* = .010), LN activity index (OR = 1.356, 95%CI: 1.090–1.687, *p* = .006), 24 h proteinuria (OR = 1.491, 95%CI: 1.075–2.068, *p* = .017), and CRP (OR = 1.062, 95%CI: 1.017–1.108, *p* = .006) were linked with higher anxiety risk (Table 2).

**Table 2:** Factors relating to anxiety risk in LN patients by logistic regression model analysis

Items	*p* value	OR	95%CI	
			Lower	Upper
Univariate logistic regression				
Age	**.012**	1.051	1.011	1.093
Gender (Female vs. Male)	.193	4.320	0.478	39.066
Disease duration	.196	1.004	0.998	1.011
SLEDAI score	**.012**	1.127	1.027	1.237
Affected nervous system (Yes vs. No)	**.012**	6.788	1.516	30.392
Affected cardiovascular system (Yes vs. No)	.239	2.411	0.557	10.429
Affected blood system (Yes vs. No)	.608	0.741	0.236	2.329
Photoallergy (Yes vs. No)	.582	0.519	0.050	5.379
Arthritis (Yes vs. No)	.193	2.200	0.670	7.220
Alopecia (Yes vs. No)	**.047**	3.361	1.016	11.117
Rash (Yes vs. No)	.275	1.904	0.599	6.054
Oral ulcer (Yes vs. No)	.149	5.625	0.540	58.575
Velcro rales (Yes vs. No)	.999	<0.001	<0.001	<0.001
Chest tightness (Yes vs. No)	1.000	2.8×10^9^	<0.001	>2.8×10^9^
Fever (Yes vs. No)	.119	3.115	0.747	12.989
LN classification				
Class II or Class III + V or Class III or Class V	Ref.			
Class IV + V or Class IV	**.010**	6.476	1.563	26.836
LN activity index	**.006**	1.356	1.090	1.687
LN chronicity index	.128	1.379	0.912	2.087
WBC	.598	1.035	0.910	1.178
Hb	.835	0.997	0.974	1.021
Platelet	.681	1.001	0.996	1.006
ALB	.789	0.989	0.908	1.076
ALT	.420	0.988	0.958	1.018
24 h proteinuria	**.017**	1.491	1.075	2.068
Scr	.056	1.010	1.000	1.021
BUN	.058	1.098	0.997	1.209
CRP	**.006**	1.062	1.017	1.108
ESR	.158	1.014	0.995	1.033
C3	.107	0.139	0.013	1.528
C4	.703	0.280	<0.001	192.250
IgA	0.548	1.234	0.622	2.450
IgG	0.665	1.017	0.944	1.095
IgM	.108	2.069	0.854	5.013
ANA (Positive vs. Negative)	.999	<0.001	<0.001	<0.001
Anti‐dsDNA (Positive vs. Negative)	.666	1.289	0.408	4.077
Anti‐Sm (Positive vs. Negative)	.565	1.488	0.384	5.770
Anti‐SSA (Positive vs. Negative)	.149	0.401	0.116	1.386
Anti‐SSB (Positive vs. Negative)	.275	0.289	0.031	2.685
Anti‐nRNP (Positive vs. Negative)	.666	0.776	0.245	2.453
Multivariate logistic regression				
Age	**.009**	1.154	1.037	1.285
LN activity index	**.020**	1.902	1.107	3.267
Alopecia (Yes vs. No)	**.023**	15.779	1.466	169.888
24 h proteinuria	**.044**	2.131	1.022	4.446
CRP	**.049**	1.075	1.000	1.155

Abbreviations: ALB, albumin; ALT, alanine aminotransferase; ANA, anti‐nuclear antibody; anti‐dsDNA, anti‐double‐stranded DNA antibody; anti‐Sm, anti‐Smith antibody; anti‐SSA, anti‐Sjögren's‐syndrome‐related antigen A antibody; anti‐SSB, anti‐Sjögren's‐syndrome‐related antigen B antibody; anti‐nRNP, anti‐nuclear ribonucleoprotein antibody; BUN, blood urea nitrogen; C3, complement component 3; C4, complement component 4; CI, confidence interval; CRP, C‐reactive protein; ESR, erythrocyte sedimentation rate; Hb, hemoglobin; IgA, immunoglobulin A; IgG, immunoglobulin G; IgM, immunoglobulin M; LN, lupus nephritis; OR, odds ratio; Scr, serum creatinine; SLEDAI, systemic lupus erythematosus disease activity index; WBC, white blood cell.

Furthermore, multivariate logistic regression revealed that age (OR = 1.154, 95%CI: 1.037–1.285, *p* = .009), LN activity index (OR = 1.902, 95%CI: 1.107–3.267, *p* = .020), alopecia (Yes vs. No) (OR = 15.779, 95%CI: 1.466–169.888, *p* = .023), 24 h proteinuria (OR = 2.131, 95%CI: 1.022–4.446, *p* = .044), and CRP (OR = 1.075, 95%CI: 1.000–1.155, *p* = .049) were independently correlated with higher anxiety risk.

The correlation of HADS scores with LN classification was also determined, which disclosed that HADS‐A score was correlated with LN classification (*p* = .021, Figure S1A), while HADS‐D score did not associate with LN classification (*p* = .365, Figure S1B).

### 3.4 Factors relating to depression risk in LN patients

Univariate logistic regression disclosed that age (OR = 1.073, 95%CI: 1.028–1.121, *p* = .001), disease duration (OR = 1.010, 95%CI: 1.001–1.018, *p* = .024), fever (Yes vs. No) (OR = 5.412, 95%CI: 1.017–28.791, *p* = .048), and LN activity index (OR = 1.258, 95%CI: 1.033–1.532, *p* = .022) were associated with higher depression risk (Table 3). Besides, complement component 3 was linked with decreased depression risk (OR = 0.097, 95%CI: 0.010–0.974, *p* = .047).

**Table 3:** Factors relating to depression risk in LN patients by logistic regression model analysis

Items	*P* value	OR	95%CI	
			Lower	Upper
Univariate logistic regression				
Age	**.001**	1.073	1.028	1.121
Gender (Female vs. Male)	.071	7.579	0.839	68.461
Disease duration	**.024**	1.010	1.001	1.018
SLEDAI score	.200	1.056	0.972	1.146
Affected nervous system (Yes vs. No)	.099	3.451	0.793	15.011
Affected cardiovascular system (Yes vs. No)	.278	2.316	0.509	10.543
Affected blood system (Yes vs. No)	1.000	1.000	0.330	3.033
Photoallergy (Yes vs. No)	.320	0.306	0.030	3.159
Arthritis (Yes vs. No)	.242	2.020	0.623	6.557
Alopecia (Yes vs. No)	.564	1.397	0.449	4.350
Rash (Yes vs. No)	.572	1.379	0.453	4.197
Oral ulcer (Yes vs. No)	.320	3.273	0.317	33.837
Velcro rales (Yes vs. No)	1.000	1.000	0.059	16.928
Chest tightness (Yes vs. No)	1.000	1.7×10^9^	<0.001	.
Fever (Yes vs. No)	**.048**	5.412	1.017	28.791
LN classification				
Class II or Class III + V or Class III or Class V	Ref.			
Class IV + V or Class IV	.251	1.962	0.621	6.193
LN activity index	**.022**	1.258	1.033	1.532
LN chronicity index	.845	1.039	0.707	1.527
WBC	.643	0.970	0.853	1.104
Hb	.925	1.001	0.978	1.024
Platelet	.279	0.997	0.992	1.002
ALB	.920	1.004	0.925	1.090
ALT	.731	0.996	0.971	1.021
24 h proteinuria	.258	1.185	0.883	1.589
Scr	.088	1.010	0.999	1.022
BUN	.058	1.111	0.997	1.238
CRP	.069	1.036	0.997	1.076
ESR	.710	0.997	0.979	1.015
C3	**.047**	0.097	0.010	0.974
C4	.712	0.310	0.001	154.139
IgA	.812	1.084	0.559	2.101
IgG	.156	1.058	0.979	1.143
IgM	.142	2.001	0.792	5.055
ANA (Positive vs. Negative)	.999	<0.001	<0.001	<0.001
Anti‐dsDNA (Positive vs. Negative)	.396	1.625	0.530	4.984
Anti‐Sm (Positive vs. Negative)	.733	1.263	0.330	4.837
Anti‐SSA (Positive vs. Negative)	.357	0.561	0.164	1.918
Anti‐SSB (Positive vs. Negative)	.115	6.000	0.647	55.661
Anti‐nRNP (Positive vs. Negative)	.396	0.615	0.201	1.887
Multivariate logistic regression				
Age	**.001**	1.097	1.040	1.157
LN activity index	**.009**	1.451	1.096	1.923

Abbreviations: ALB, albumin; ALT, alanine aminotransferase; ANA, anti‐nuclear antibody; anti‐dsDNA, anti‐double‐stranded DNA antibody; anti‐Sm, anti‐Smith antibody; anti‐SSA, anti‐Sjögren's‐syndrome‐related antigen A antibody; anti‐SSB, anti‐Sjögren's‐syndrome‐related antigen B antibody; anti‐nRNP, anti‐nuclear ribonucleoprotein antibody; BUN, blood urea nitrogen; C3, complement component 3; C4, complement component 4; CI, confidence interval; CRP, C‐reactive protein; ESR, erythrocyte sedimentation rate; Hb, hemoglobin; IgA, immunoglobulin A; IgG, immunoglobulin G; IgM, immunoglobulin M; LN, lupus nephritis; OR, odds ratio; Scr, serum creatinine; SLEDAI, systemic lupus erythematosus disease activity index; WBC, white blood cell.

Moreover, multivariate logistic regression revealed that age (OR = 1.097, 95%CI: 1.040–1.157, *p* = .001) and LN activity index (OR = 1.451, 95%CI: 1.096–1.923, *p* = .009) were independently correlated with higher depression risk.

## 4 DISCUSSION

Anxiety and depression are frequently occurred in autoimmune diseases; meanwhile, some previous studies disclose that the frequency of anxiety and depression is high in several autoimmune diseases^12^, ^19^, ^20^; one study reports that anxiety and depression rate is 33.8% and 36.9% (evaluated by HADS) in Sjogren's syndrome patients.^20^ Another study illustrates that the frequency of anxiety and depression is 27.1% and 40.0%, separately in psoriatic arthritis patients.^19^ What's more, a systematic review discloses that anxiety and depression have a prevalence of 40.0% and 30%, respectively in SLE patients.^13^ Whereas research about the prevalence of anxiety and depression in LN remains elusive. Only one study indicates that patients with mental health problems might be altered during the treatment of LN; in detail, anxiety, and depression could be alleviated after the induction treatment of LN patients.^21^ In the current study, LN patients had higher anxiety and depression scores compared with non‐LN SLE patients and HCs. Meanwhile, LN patients had higher anxiety and depression rates compared with non‐LN SLE patients and HCs. Possible explanations could be that: (1) LN patients might face several bleaker events such as pain, disability, discrimination, fear of mortality, and social stress, which might result in psychological problems. (2) The occurrence of LN is accompanied by the recruitment of proinflammation cytokines, which are reported to be closely related to anxiety and depression, subsequently, anxiety and depression have high frequencies in LN patients.^22^, ^23^ HADS scale was applied in this study to assess the anxiety and depression, but not other scales such as the Zung scale and Hamilton scale. The reason is as follows: other scales are either too complex or the requirement of the psychiatrist to assess, while the HADS scale is simple and convenient. Therefore, HADS scale is applied in our study.

In the current study, we also evaluated the independent factors for anxiety and depression risks in LN patients: the multivariate logistic regression disclosed that age, LN activity index, alopecia, 24 h proteinuria, and CRP were independently correlated with higher anxiety risk, meanwhile, age and LN activity index were independently correlated with higher depression risk. Possible explanations could be that: (1) More severe renal involvement could deteriorate the social and daily life of LN patients, which might cause anxiety and depression, hence the LN activity index and 24 h proteinuria relate to anxiety and depression independently in LN patients.^24^, ^25^ (2) Aging usually relates to increased disability, more complications, and increased costs for daily management. Thereby age is an independent factor for anxiety and depression in LN patients.^26^ (3) Alopecia could have a negative impact on personal appearance, which might influence an individual's career and daily social, and this might subsequently induce the occurrence of anxiety; therefore, alopecia could be an independent factor for anxiety.^27^, ^28^ (4) Similar to previous studies, long‐term systematic inflammation could harm mental health, thus CRP exhibits to be an independent factor for anxiety.^29^, ^30^

Despite the innovation of the current study, some limitations existed in the current study: (1) The current study was a single‐centered study, which might result in less generalizability of our results. (2) The HADS scores for evaluating anxiety and depression were a subjective, self‐assessed questionnaire, which might exist an assessment bias. (3) This study merely enrolled 50 LN patients; thus, the sample size was relatively small. (4) The current study did not investigate the underlying pathogenesis of anxiety and depression in LN patients, which needed to be further explored.

In conclusion, anxiety and depression are highly prevalent, which link to aging, alopecia, inflammation, and severe renal involvement in LN patients.

## AUTHOR CONTRIBUTIONS

Ying Hu conceived the study, collected and analyzed data, and wrote the first draft. Ge Zhan analyzed data, wrote and edited the manuscript. All authors contributed to the article and approved the submitted version.

## CONFLICTS OF INTEREST

The authors declare no conflicts of interest.

## ETHICS STATEMENT

This study was approved by Institutional Review Board, and each subject signed an informed consent.

## Supporting information

Figurementary Figure 1.Correlation of the HADS scores with the LN classification. Correlation of the HADS‐A (**A**) and HADS‐D (**B**) scores with the LN classification.

## Data Availability

The datasets used and/or analysed during the current study are available from the corresponding author on reasonable request.
