# Supplementary figures and images for: Anxiety and depression prevalence and their risk factors in lupus nephritis patients: A case–control study
Source: Immun Inflamm Dis. 2022 Aug 22;10(9):e689. doi: 10.1002/iid3.689 (PMC9394231; doi:10.1002/iid3.689)

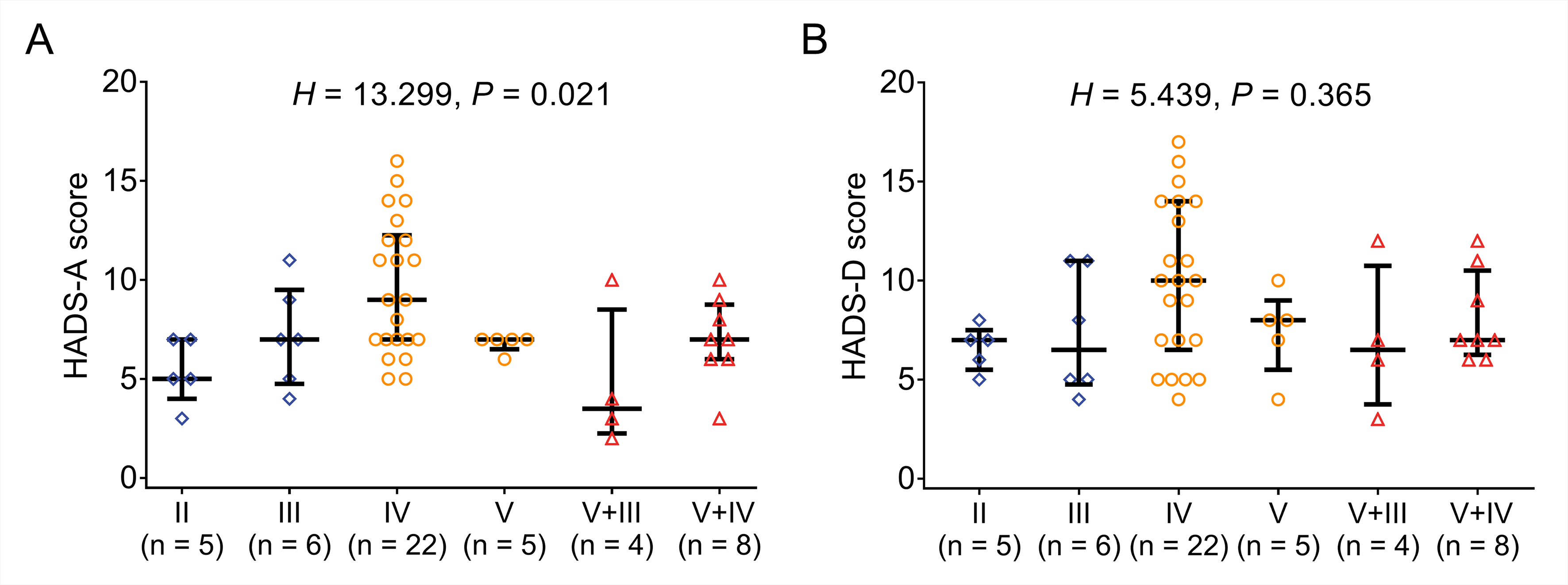

Supplement: Supplementary file 1 — Figurementary Figure 1.Correlation of the HADS scores with the LN classification. Correlation of the HADS‐A (A) and HADS‐D (B) scores with the LN classification. [file IID3-10-e689-s001.tif]
